# Diagnostic Pitfall: Mixed Connective Tissue Disease‐Associated Pulmonary Hypertension Presenting With Postpartum Right‐Sided Heart Failure in a 21‐Year‐Old—Case Report

**DOI:** 10.1002/ccr3.73891

**Published:** 2026-11-07

**Authors:** Sai Santhosha Mrudula Alla, Moksh Sanjay Khetan, Deekshitha Alla, Sri Lakshmi Ganguru, Srinidhi Appala, Krithi Sree, Riva Jayesh Shah, Aryan Seth, Nishant Anand Rawtani, Mohammed Raihanuddin Aamir, Nyassiri Warai Emmanuel

**Affiliations:** ^1^ Andhra Medical College Visakhapatnam Andhra Pradesh India; ^2^ Vedantaa Institute of Medical Sciences Dahanu India; ^3^ Department of Pediatrics Andhra Medical College Visakhapatnam Andhra Pradesh India; ^4^ NRI Medical College and General Hospital Guntur India; ^5^ All India Institute of Medical Sciences Bhopal Bhopal India; ^6^ ESIC MC and Hospital Chennai India; ^7^ Kasturba Medical College Mangalore India; ^8^ Internal Medicine Kasturba Medical College Mangalore India; ^9^ Lokmanya Tilak Municipal Medical College and General Hospital Mumbai India; ^10^ Shadan Institute of Medical Sciences, Teaching Hospital and Research Center Hyderabad India; ^11^ Faculty of Medicine and Biomedical Sciences University of Garoua Garoua Cameroon

**Keywords:** anasarca, connective tissue disease, interstitial syndrome, post‐partum heart failure, systemic disease

## Abstract

Mixed connective tissue disease is a multisystem autoimmune condition characterized by overlapping clinical and serological features related to anti‐U1‐RNP antibodies of at least two connective tissue diseases. It may present with myocarditis, pericarditis, pulmonary arterial hypertension, and, less commonly, heart failure. We present a 21‐year‐old female with bilateral foot swelling, cough, and shortness of breath 3 months after a vaginal delivery. On examination, she was hypotensive (90/70 mmHg), with an elevated jugular venous pressure, a pulmonary systolic murmur with loud P2, and a tricuspid regurgitation murmur. Bronchial breath sounds and fine crepitations were present in both lower lung fields. She was evaluated initially for suspected postpartum cardiomyopathy. Transthoracic echocardiography was suggestive of a probable moderate to severe PAH. High‐resolution computed tomography showed bilateral moderate pleural effusions, honeycombing with partial atelectasis of the underlying lung, and cardiomegaly. Computed Tomography Pulmonary Angiography (CTPA) showed findings likely consistent with probable mild pulmonary hypertension. An antinuclear antibody panel was performed and was positive for anti‐U1‐RNP, SS‐A, SS‐B, and Jo‐1 antibodies. She was started on Lasix 40 mg, Aldactone 25 mg, lisinopril 10 mg, and heparin. Treatment with 20 mg of prednisolone was started and gradually tapered. Her anasarca and dyspnea improved, and she was discharged with follow‐up arranged with cardiology and rheumatology. Timely identification of MCTD‐associated pulmonary hypertension and its cardiovascular complications is essential and allows early commencement of treatment, which further prevents the patients from developing heart failure.

## Key Clinical Message

This is an interesting and clinically relevant case report describing post‐partum heart failure initially suspected to be post‐partum cardiomyopathy but later attributed to mixed connective tissue disease with pulmonary hypertension and right‐heart involvement. The manuscript highlights an important diagnostic pitfall and provides a useful clinical learning point.

**Abbreviations**
2D echo: two‐dimensional echocardiogram
ANA: antinuclear antibody
anti‐SMN: antibodies against the survival motor neuron complex
anti‐U1‐RNP: anti‐U1 ribonucleoprotein antibody
CD4^+^ cells: cluster of differentiation 4‐positive T‐helper cells
CT: computed tomography
CTPA: computed tomography pulmonary angiography
CTD‐PAH: connective tissue disease‐associated pulmonary arterial hypertension
HRCT: high‐resolution computed tomography
IL‐1: interleukin 1
IL‐6: interleukin 6
Jo‐1: histidyl‐tRNA synthetase antibody
MCTD: mixed connective tissue disease
mPAP: mean pulmonary artery pressure
NT‐proBNP: N‐Terminal pro‐B‐type Natriuretic Peptide
NYHA: New York Heart Association
PAH: pulmonary artery hypertension
PCWP: pulmonary capillary wedge pressure
PSM: pansystolic murmur
PVR: pulmonary vascular resistance
RHF: right heart failure
RVSP: right ventricular systolic pressure
SLE: systemic lupus erythematosus
SOB: shortness of breath
SS: systemic sclerosis
SS‐A: Sjögren's Syndrome‐related antigen A
SS‐B: Sjögren's Syndrome‐related antigen B
TRV: tricuspid regurgitation jet velocity

## 1 Introduction

Mixed connective tissue disease (MCTD) is an uncommon systemic autoimmune condition characterized by features of at least two connective tissue diseases, most commonly rheumatoid arthritis, polymyositis, dermatomyositis, systemic lupus erythematosus (SLE), and systemic sclerosis (SS) [1]. Raynaud phenomenon, puffy fingers, and hand edema are typical clinical features of MCTD, which is also characterized by high titers of anti‐U1‐RNP antibodies [2]. Other immunological markers, such as high‐titer speckled pattern antinuclear antibody (ANA), rheumatoid factor, and anti‐cyclic citrullinated peptide, can also be positive [3, 4]. Another potential marker for MCTD is antibodies against the survival motor neuron complex (anti‐SMN), which have been linked to increased prevalence of pulmonary arterial hypertension (PAH) and interstitial lung disease [5]. Diagnosis is based on immunological markers and overlapping presentations such as synovitis, myositis, pleuritis, pericarditis, leukopenia, esophageal dysmotility, or interstitial lung disease, despite the absence of distinctive clinical signs [2]. Recent proposals emphasize organ involvement, such as PAH, trigeminal neuropathy, or aseptic meningitis. According to Orphanet, a registry for rare diseases, the estimated prevalence of MCTD in the general population is 3.8 per 100,000 worldwide [6]. In addition to myocarditis and pericarditis, PAH is a well‐recognized complication of MCTD and a major contributor to right heart failure (RHF) [7, 8]. We present a young woman with RHF who was initially suspected of having postpartum cardiomyopathy and then discovered to have MCTD‐associated pulmonary artery hypertension, presenting with postpartum right‐sided heart failure. A resource‐limited tertiary center in India established the diagnosis.

## 2 Case History/Examination

A 21‐year‐old woman presented with bilateral foot swelling, cough, and shortness of breath (SOB) for 2 weeks. She had delivered vaginally 3 months earlier. She denied any chills or fever. Dyspnea was insidious in onset, initially exertional but later present at rest. She also experienced orthopnea, fatigue, and paroxysmal nocturnal dyspnea.

On examination, she was hypotensive with a blood pressure of 90/70 mmHg, and the rest of her vitals were stable. On general examination, the only finding was bilateral pitting pedal edema. On cardiovascular examination, jugular venous pressure was elevated to 11 cm H_2_O, with a loud P2 and a pansystolic murmur at the tricuspid area. There were bronchial breath sounds and fine crepitations in the bilateral lower lung fields.

## 3 Methods

Laboratory investigations revealed a hemoglobin of 12.6 g/dL, a hematocrit of 38.7%, a leukocyte count of 3600/μL, and a platelet count of 121,000/μL. The renal and liver function tests were within normal limits. Table 1 summarizes the laboratory investigations. An ANA profile was performed and showed positive results for anti‐U1‐RNP, SS‐A, SS‐B, and Jo‐1 antibodies. Table 2 shows the patient's ANA profile. Additional autoimmune and rheumatologic investigation results beyond the reported antibody profile were unavailable in the records reviewed, limiting further characterization of the underlying connective tissue disease. The chest X‐ray revealed cardiomegaly, and the electrocardiogram showed a heart rate of 96 beats per minute and right axis deviation.

**TABLE 1:** Hematological parameters in the patient.

Parameter	Most recent value	Reference ranges
Hemoglobin (Hb)	12.6 g/dL (Low‐normal)	12–16 g/dL
Total leukocyte count (TLC)	**3600/μL**	4500–11,000/μL
Platelet count	**1.21 lakh/μL**	150,000–400,000/μL
Packed cell volume (PCV)	38%	36%–48%
Neutrophils	50%	40%–60%
Lymphocytes	45%	20%–40%
Eosinophils	4%	1%–4%
Monocytes	1%	2%–8%
Blood urea	22.4 mg/dL	Verify laboratory range for urea, not BUN
Serum creatinine	1.1 mg/dL	0.6–1.3 mg/dL
Total bilirubin	1.0 mg/dL	0.1–1.2 mg/dL
Direct bilirubin	0.3 mg/dL	< 0.3 mg/dL
Indirect bilirubin	0.7 mg/dL	Verify laboratory range
SGPT (ALT)	52.3 IU/L	4–36 IU/L
SGOT (AST)	29.8 IU/L	8–33 IU/L
Alkaline phosphatase (ALP)	**131 IU/L**	20–130 IU/L
Total protein	6.7 g/dL	6.0–8.3 g/dL
Serum calcium (Ca^2+^)	9.1 mg/dL	8.5–10.2 mg/dL
Serum magnesium (Mg^2+^)	2.4 mg/dL	1.7–2.2 mg/dL
Serum uric acid	**8.1 mg/dL**	Verify the laboratory's adult female range
Ph	5.0	Confirm specimen before adding range

*Note:* The bold values represents results that are not within the normal range.

**TABLE 2:** ANA profile of the patient.

Parameter	Results
Anti‐U1‐RNP	**Positive**
SS‐A/Ro60	**Positive**
SS‐B (La)	**Positive (++)**
Jo‐1	**Positive**
CENP B	Borderline
PCNA	Borderline
Control band	**Strong positive (+++)**
U1RNP	**Positive**

*Note:* The bold values represents results that are not within the normal range.

The 2D echo showed significant right heart involvement with a dilated right atrium and right ventricle, with moderate to severe tricuspid regurgitation. The tricuspid regurgitation jet velocity (TRV) was elevated to 3.5 m/s, accompanied by an increased right ventricular systolic pressure (RVSP) of approximately 50 mmHg, which is suggestive of probable moderate to severe PAH based on the echocardiographic results. Right heart catheterization (RHC) was not performed due to resource limitations. The inferior vena cava appeared dilated, indicating elevated right atrial pressure. Mild pericardial effusion was also noted. Left ventricular systolic function was preserved, with an ejection fraction of around 60%. No regional wall motion abnormalities were seen.

Computed tomography (CT) chest revealed cardiomegaly, honeycombing in the lungs, and bilateral pleural effusions, suggesting PAH and heart failure. Figure 1 shows the axial view of the chest, and Figure 2 shows the coronal view of the chest. Computed tomography pulmonary angiogram (CTPA) revealed a primary pulmonary artery diameter of 28 mm, which is a standard caliber and suggests probable mild pulmonary hypertension. However, CTPA is not a reliable tool for assessing PAH severity compared to electrocardiographic measurement and was primarily utilized to exclude acute pulmonary embolism in the postpartum period. The left and right pulmonary artery diameters were 17 and 16.5 mm, respectively. Bilateral pleural effusion was noted, along with pleural separation on the right and left sides, measuring 33 and 14 mm (right greater than left). Right atrial enlargement was also seen. High‐resolution computed tomography (HRCT) of the chest revealed bilateral moderate pleural effusions, with honeycombing and partial atelectasis of the underlying lung. Cardiomegaly with mild pericardial effusion was also noted.

**FIGURE 1:**
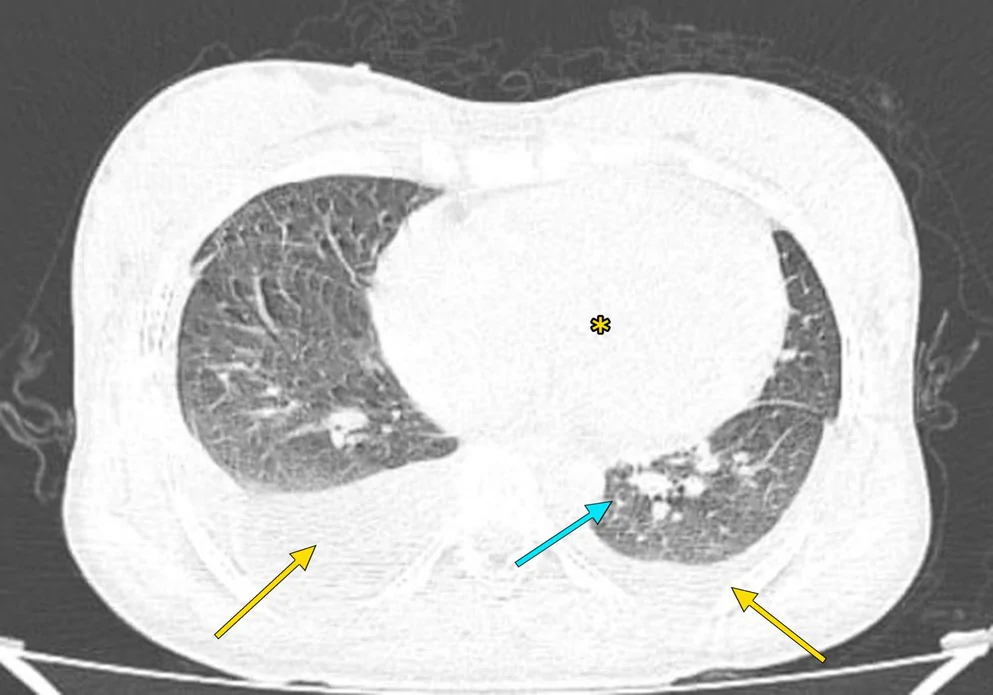
Axial view of the chest. This axial view shows bilateral moderate pleural effusions, honeycombing with partial atelectasis of the underlying lung, and cardiomegaly.

**FIGURE 2:**
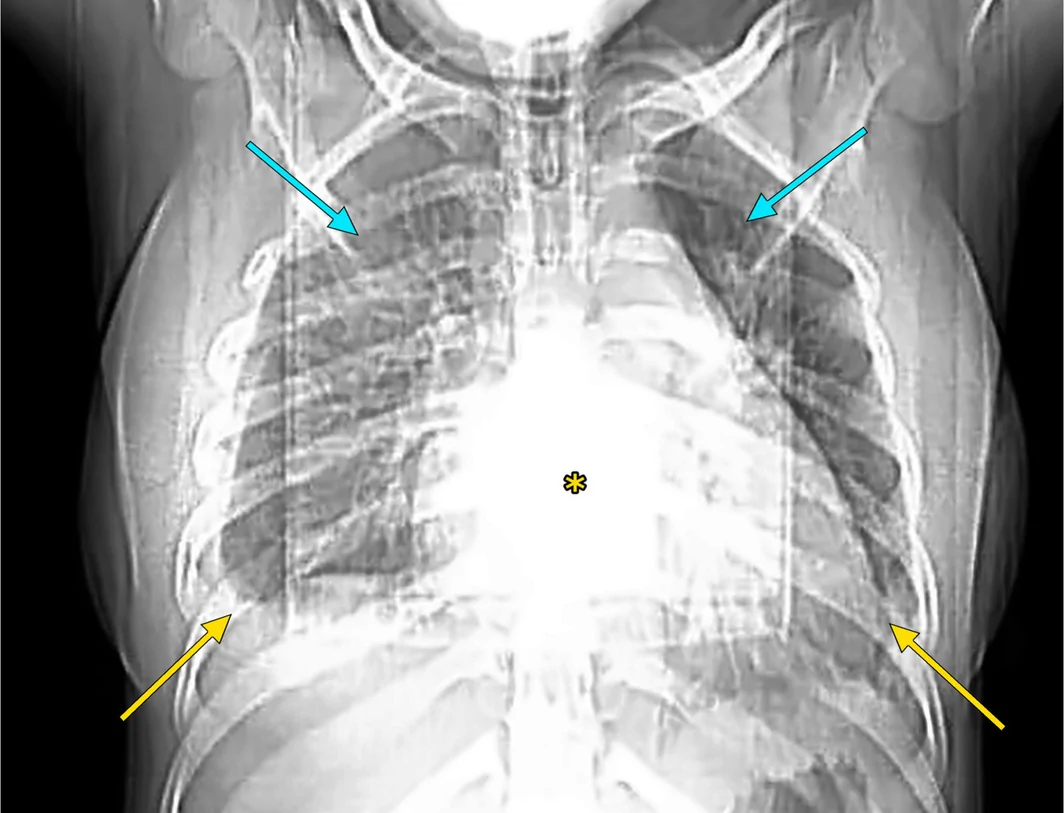
Coronal view of the chest. This coronal view shows bilateral moderate pleural effusions, cardiomegaly, vascular redistribution to the apices with no apparent signs of pulmonary embolism.

PPCM and acute pulmonary embolism were considered in the evaluation of postpartum heart failure. PPCM was not supported by the preserved LVEF of approximately 60% and the predominant right‐sided echocardiographic abnormalities. CTPA showed no evidence of acute pulmonary embolism. Anti‐U1‐RNP positivity, leukopenia, thrombocytopenia, and interstitial lung abnormalities supported an underlying connective tissue disease. However, the available findings did not establish whether pulmonary hypertension predominantly reflected connective tissue disease‐associated pulmonary arterial vasculopathy, interstitial lung disease, or a combination of these mechanisms. RHC was unavailable, limiting hemodynamic classification.

## 4 Conclusion and Results

Initially, the patient was diagnosed with postpartum cardiomyopathy; however, subsequent investigations established heart failure secondary to MCTD. The diagnosis of MCTD‐associated pulmonary hypertension was validated using the Sharp criteria [9]. Table 3 shows the major and minor Sharp criteria and the prerequisites the patient meets. The treating team used the Sharp criteria because they were readily available in the local clinical setting. Formal assessment using the Alarcón‐Segovia or Kasukawa criteria was not performed; therefore, fulfillment of these alternative classification criteria could not be established, which represents a limitation of this report.

**TABLE 3:** Sharp criteria for which the patient is positive.

Major criteria	
Pulmonary involvement	**Yes**
Esophageal hypomotility or Raynaud's phenomenon	No
Myositis	No
Swollen hands	**Yes**
Serology	**Yes**

Minor criteria	
Alopecia	No
Anemia	No
Pericarditis	**Yes**
Pleuritis	**Yes**
Leukopenia	**Yes**
Myositis	No
Malar rash	No
Thrombocytopenia	**Yes**
History of swollen hands	**Yes**
Trigeminal neuropathy	No
Arthritis	No

*Note:* The bold values represents results that are not within the normal range.

Immediately following admission, she was started on Lasix 40 mg, Aldactone 25 mg, lisinopril 10 mg, and heparin. Treatment with 20 mg of prednisolone was started and gradually tapered. The steroids were initiated to target the underlying systemic inflammatory process of the MCTD. Her anasarca and dyspnea improved, following which she was discharged and instructed to follow up with the cardiologist and rheumatologist for further management.

After treatment, the patient improved clinically, with reduced anasarca and dyspnea. She was subsequently discharged in stable condition, with recommendations to continue follow‐up with cardiology and rheumatology services. However, the patient was lost to follow‐up after discharge, and we could not assess her long‐term clinical outcomes, treatment adherence, or subsequent cardiopulmonary status.

## 5 Discussion

Peripartum cardiomyopathy (PPCM) was initially suspected because the patient presented 3 months after delivery with new‐onset progressive dyspnea, orthopnea, paroxysmal nocturnal dyspnea, peripheral edema, and elevated jugular venous pressure. The postpartum timing and clinical features of heart failure prompted this initial diagnostic consideration. However, echocardiography demonstrated preserved left ventricular systolic function, with an LVEF of approximately 60%, which did not meet the defining criterion of left ventricular systolic dysfunction with an LVEF < 45% for PPCM. Instead, the predominant abnormalities were right atrial and right ventricular dilatation, moderate‐to‐severe tricuspid regurgitation, an elevated tricuspid regurgitation velocity of 3.5 m/s, and a dilated inferior vena cava. Although right ventricular involvement can accompany PPCM, the predominant right‐sided abnormalities with preserved LVEF favored PAH‐associated right‐sided heart failure over PPCM.

MCTD causes a spectrum of symptoms through different pathways. The first pathway involves the production of immunological complexes, which can trigger the complement cascade and lead to tissue damage and inflammation, ultimately resulting in interstitial lung disease, myositis, and arthritis [10]. The second pathway involves the altered U1 70 kDa protein, which appears on the surface of dying cells. Antigen‐presenting cells can detect this protein and present it to T cells, which may then help B cells produce antibodies against U1‐RNP. Anti‐U1‐RNP can stimulate both autoreactive CD4^+^ cells and mononuclear cells, increasing IL‐6 and IL‐1 production. In addition, autoantibodies can directly damage endothelial cells. It can stimulate the generation of endothelial and intercellular leukocyte adhesion molecule‐1, which functions as an anti‐endothelial cell agent. This involvement affects both the pulmonary and systemic vascular systems, often manifesting as PAH or Raynaud's phenomenon [11].

In MCTD patients, cardiac involvement occurs in approximately 65% of cases, but only 25% are symptomatic in reported cohorts [7]. The typical cardiac manifestations include pericarditis, PAH, and diastolic dysfunction; however, certain other conditions, like mitral valve prolapse and cardiac conduction abnormalities, have also been documented. PAH and pericardial or pleural effusions are leading contributors to morbidity and mortality. Definitive information regarding the underlying cellular process of cardiac involvement is scarce in the literature. This case primarily presented with suspected PAH and heart failure as manifestations of MCTD. This patient had a loud P2, bilateral pedal and facial edema, cough, SOB, and a pansystolic murmur in the tricuspid region, which could indicate tricuspid regurgitation or probable PAH [7]. These symptoms suggest predominant cardiovascular involvement. These results are consistent with infrequent instances of MCTD that primarily manifest as cardiovascular symptoms [12]. A known complication of MCTD, PAH, is probably the cause of the loud P2 and PSM, which show significant right heart strain [13]. This case underscores the need for early cardiac evaluation in MCTD patients, as cardiovascular symptoms can dominate and progress rapidly. Multidisciplinary management, involving rheumatologists and cardiologists, is crucial for optimizing outcomes.

MCTD is diagnosed clinically, supported by serology. The two most widely accepted criteria are the Alarcón‐Segovia and Kasukawa criteria, both of which require anti‐U1 RNP antibodies and clinical features overlapping with SLE, SS, and polymyositis [14, 15]. Additional serological markers include ANA with a speckled pattern, cytopenias, elevated muscle enzymes (in inflammatory myopathy), and overlapping antibodies such as SS‐A, SS‐B, and Jo‐1 [16, 17]. First‐line screening for suspected PAH includes transthoracic echocardiography to evaluate TRV, right heart size, and RV function [18]. HRCT is a valid imaging modality for assessing pleural effusions, interstitial lung disease, and atelectasis. RHC remains the diagnostic gold standard, confirming pre‐capillary pulmonary hypertension by demonstrating a mean pulmonary artery pressure (mPAP) > 20 mmHg, pulmonary vascular resistance (PVR) > 3 Wood units, and a pulmonary capillary wedge pressure (PCWP) ≤ 15 mmHg [19]. However, this could not be performed in this case due to limited available resources.

Concomitant anti‐Jo‐1 positivity, together with the interstitial lung abnormalities, raises the possibility of antisynthetase syndrome or an overlapping connective tissue disease. However, additional rheumatologic investigation results and documentation of a targeted assessment for these conditions were unavailable. Consequently, these possibilities could not be conclusively evaluated, representing a limitation of this report.

Treatment is highly individualized, with immunosuppressive agents such as high‐dose corticosteroids, hydroxychloroquine, mycophenolate mofetil, and cyclophosphamide forming the backbone of therapy [20]. In resistant cases, rituximab can be considered [21]. Advanced PAH requires targeted vasodilator therapy, including endothelin receptor antagonists, phosphodiesterase‐5 inhibitors, and prostacyclin analogs [22]. Early multidisciplinary involvement and referral for lung transplantation are essential in refractory cases [23]. The NYHA functional class and exercise tolerance influence prognosis [24]. Elevated NT‐proBNP or increasing autoantibody titers may reflect active disease [25]. Although anti‐U1 RNP is a hallmark, some studies suggest it may confer better survival in CTD‐PAH, possibly reflecting a unique disease phenotype [26]. While historically poorer than idiopathic PAH, survival has improved with early detection and targeted treatment [27].

One of the biggest challenges was the delay in diagnosis, largely due to delayed ANA profiling. The patient was initially diagnosed with postpartum cardiomyopathy, but later, with 2D echo findings, an ANA profile was done, which turned out to be positive. However, treatment for both conditions overlapped, and immunosuppression was specific to MCTD. Therefore, she received treatment for heart failure promptly. However, immunosuppressive therapy was delayed because the diagnosis was delayed. The patient was also referred to an external rheumatology service for follow‐up due to institutional restraints.

Timely identification of MCTD and its cardiovascular complications is essential and allows early commencement of treatment, which further prevents patients from developing heart failure.

## Author Contributions

**Sai Santhosha Mrudula Alla:** conceptualization, data curation, formal analysis, writing – original draft, writing – review and editing. **Moksh Sanjay Khetan:** writing – original draft, writing – review and editing. **Deekshitha Alla:** writing – original draft, writing – review and editing. **Sri Lakshmi Ganguru:** writing – original draft, writing – review and editing. **Srinidhi Appala:** writing – original draft, writing – review and editing. **Krithi Sree:** writing – original draft, writing – review and editing. **Riva Jayesh Shah:** writing – original draft, writing – review and editing. **Aryan Seth:** writing – original draft, writing – review and editing. **Nishant Anand Rawtani:** writing – original draft, writing – review and editing. **Mohammed Raihanuddin Aamir:** writing – original draft, writing – review and editing. **Nyassiri Warai Emmanuel:** writing – original draft, writing – review and editing.

## Funding

The authors have nothing to report.

## Ethics Statement

Ethical approval was not required because we did not use the patient's identifying information, and we reported this case in accordance with the TITAN guidelines.

## Consent

We obtained written informed consent from the patient for publication and any accompanying images.

## Conflicts of Interest

The authors declare no conflicts of interest.

## Data Availability

The clinical data supporting this case report are available from the corresponding author upon reasonable request for review by the journal editors and reviewers, subject to patient confidentiality and applicable institutional policies.

## References

[1] G. C. Sharp , W. S. Irvin , E. M. Tan , R. G. Gould , and H. R. Holman , “Mixed Connective Tissue Disease—An Apparently Distinct Rheumatic Disease Syndrome Associated With a Specific Antibody to an Extractable Nuclear Antigen (ENA),” American Journal of Medicine 52, no. 2 (1972): 148–159, 10.1016/0002-9343(72)90064-2. 4621694

[2] R. Gunnarsson , S. O. Hetlevik , V. Lilleby , and Ø. Molberg , “Mixed Connective Tissue Disease,” Best Practice & Research. Clinical Rheumatology 30, no. 1 (2016): 95–111, 10.1016/j.berh.2016.03.002. 27421219

[3] Y. Takasaki , K. Yamanaka , C. Takasaki , et al., “Anticyclic Citrullinated Peptide Antibodies in Patients With Mixed Connective Tissue Disease,” Modern Rheumatology 14, no. 5 (2004): 367–375, 10.1007/s10165-004-0325-2. 17143695

[4] I. García‐de la Torre , M. Salazar‐Páramo , and G. Salmón‐de la Torre , “Mixed Connective Tissue Disease. A Clinico‐Serological Study of 17 Cases,” Molecular Biology Reports 23, no. 3–4 (1996): 153–157, 10.1007/BF00351163. 9112223

[5] Y. Todoroki , M. Satoh , S. Kubo , et al., “Anti‐Survival Motor Neuron Complex Antibodies as a Novel Biomarker for Pulmonary Arterial Hypertension and Interstitial Lung Disease in Mixed Connective Tissue Disease,” Rheumatology 63, no. 4 (2024): 1068–1075, 10.1093/rheumatology/kead341. 37421400

[6] Orphanet , “Mixed Connective Tissue Disease,” Orphanet, accessed July 19, 2025, https://www.orpha.net/en/disease/detail/809.

[7] P. Ungprasert , T. Wannarong , T. Panichsillapakit , et al., “Cardiac Involvement in Mixed Connective Tissue Disease: A Systematic Review,” International Journal of Cardiology 171, no. 3 (2014): 326–330, 10.1016/j.ijcard.2013.12.079. 24433611

[8] Y. K. Sung and L. Chung , “Connective Tissue Disease‐Associated Pulmonary Arterial Hypertension,” Rheumatic Disease Clinics of North America 41, no. 2 (2015): 295–313, 10.1016/j.rdc.2015.01.003. 25836644

[9] G. C. Sharp , Diagnostic Criteria for Classification of MCTD (Mixed Connective Tissue Disease and Antinuclear Antibodies) (Elsevier, 1987), 23–32.

[10] C. A. Ferrara , G. La Rocca , G. Ielo , A. Libra , and G. Sambataro , “Towards Early Diagnosis of Mixed Connective Tissue Disease: Updated Perspectives,” ImmunoTargets and Therapy 31 (2023): 79–89. 10.2147/ITT.S390023 PMC10387239 37525698

[11] B. Sapkota and Y. Al Khalili , “Mixed Connective Tissue Disease,” in InStatPearls (StatPearls Publishing, 2024). 31194355

[12] T. Hamana , H. Kawamori , S. Satomi‐Kobayashi , Y. Yamamoto , Y. Ikeda , and K. I. Hirata , “Fulminant Myocarditis in a Young Woman With Mixed Connective Tissue Disease: A Case Report,” European Heart Journal—Case Reports 7, no. 4 (2023): ytad174, 10.1093/ehjcr/ytad174. PMC10122413 37096149

[13] D. Ü. Cansu and C. Korkmaz , “Pulmonary Hypertension in Connective Tissue Diseases: Epidemiology, Pathogenesis, and Treatment,” Clinical Rheumatology 42, no. 10 (2023): 2601–2610, 10.1007/s10067-022-06446-y. 36396789

[14] D. Alarcón‐Segovia and M. Villarreal , “Classification and Diagnostic Criteria for Mixed Connective Tissue Disease,” in Mixed Connective Tissue Disease and Anti‐Nuclear Antibodies, ed. R. Kasukawa and G. C. Sharp (Elsevier, 1987), 33–40.

[15] R. Kasukawa , T. Tojo , and S. Miyawaki , “Preliminary Diagnostic Criteria for Classification of Mixed Connective Tissue Disease,” in Mixed Connective Tissue Disease and Anti‐Nuclear Antibodies, ed. R. Kasukawa and G. C. Sharp (Elsevier Science Publishers, 1987), 41–47.

[16] M. Mahler , P. L. Meroni , X. Bossuyt , and M. J. Fritzler , “Current Concepts and Future Directions for the Assessment of Autoantibodies to Cellular Antigens Referred to as Anti‐Nuclear Antibodies,” Journal of Immunology Research 2014 (2014): 315179, 10.1155/2014/315179. PMC4020446 24868563

[17] K. J. John , M. Sadiq , T. George , et al., “Clinical and Immunological Profile of Mixed Connective Tissue Disease and a Comparison of Four Diagnostic Criteria,” International Journal of Rheumatology 2020 (2020): 9692030, 10.1155/2020/9692030. PMC7204172 32411251

[18] R. M. Lang , L. P. Badano , V. Mor‐Avi , et al., “Recommendations for Cardiac Chamber Quantification by Echocardiography in Adults: An Update From the American Society of Echocardiography and the European Association of Cardiovascular Imaging,” Journal of the American Society of Echocardiography 28, no. 1 (2015): 1–39.e14, 10.1016/j.echo.2014.10.003. 25559473

[19] G. Simonneau , D. Montani , D. S. Celermajer , et al., “Hemodynamic Definitions and Updated Clinical Classification of Pulmonary Hypertension,” European Respiratory Journal 53, no. 1 (2019): 1801913, 10.1183/13993003.01913-2018. PMC6351336 30545968

[20] K. Chevalier , B. Thoreau , M. Michel , et al., “Treatment of Mixed Connective Tissue Disease: A Multicenter Retrospective Study,” Journal of Autoimmunity 153 (2025): 103420, 10.1016/j.jaut.2025.103420. 40245724

[21] L. Montolio‐Chiva , J. Narváez , J. J. Alegre‐Sancho , et al., “AB0594 Effectiveness of Rituximab in CSDMARDS‐Resistant Active Mixed Connective Tissue Disease,” Annals of the Rheumatic Diseases 79 (2020): 1593–1594, 10.1136/annrheumdis-2020-eular.4778.

[22] E. Zanatta , P. Polito , G. Famoso , et al., “Pulmonary Arterial Hypertension in Connective Tissue Disorders: Pathophysiology and Treatment,” Experimental Biology and Medicine 244, no. 2 (2019): 120–131, 10.1177/1535370218824101. PMC6405825 30669861

[23] M. P. George , H. C. Champion , and J. M. Pilewski , “Lung Transplantation for Pulmonary Hypertension,” Pulmonary Circulation 1, no. 2 (2011): 182–191, 10.4103/2045-8932.83455. PMC3198646 22034605

[24] R. J. Barst , L. Chung , R. T. Zamanian , M. Turner , and M. D. McGoon , “Functional Class Improvement and 3‐Year Survival Outcomes in Patients With Pulmonary Arterial Hypertension in the REVEAL Registry,” Chest 144, no. 1 (2013): 160–168, 10.1378/chest.12-2417. 23429998

[25] H. Tang , F. Lu , Y. Huang , et al., “Group‐Based Trajectory Modeling of N‐Terminal Pro‐Brain Natriuretic Peptide Levels in Pulmonary Artery Hypertension Associated With Connective Tissue Disease,” Healthcare 12, no. 16 (2024): 1633, 10.3390/healthcare12161633. PMC11354151 39201191

[26] V. Sobanski , J. Giovannelli , B. M. Lynch , et al., “Characteristics and Survival of Anti‐U1 RNP Antibody‐Positive Patients With Connective Tissue Disease‐Associated Pulmonary Arterial Hypertension,” Arthritis & Rhematology 68, no. 2 (2016): 484–493, 10.1002/art.39432. 26415038

[27] D. Khanna , C. Zhao , R. Saggar , et al., “Long‐Term Outcomes in Patients With Connective Tissue Disease‐Associated Pulmonary Arterial Hypertension in the Modern Treatment Era: Meta‐Analyses of Randomized, Controlled Trials and Observational Registries,” Arthritis and Rheumatology 73, no. 5 (2021): 837–847, 10.1002/art.41669. PMC8251834 33538058

